# Childhood Obesity: Position Statement of Polish Society of Pediatrics, Polish Society for Pediatric Obesity, Polish Society of Pediatric Endocrinology and Diabetes, the College of Family Physicians in Poland and Polish Association for Study on Obesity

**DOI:** 10.3390/nu14183806

**Published:** 2022-09-15

**Authors:** Artur Mazur, Agnieszka Zachurzok, Joanna Baran, Katarzyna Dereń, Edyta Łuszczki, Aneta Weres, Justyna Wyszyńska, Justyna Dylczyk, Ewa Szczudlik, Dorota Drożdż, Paulina Metelska, Michał Brzeziński, Agnieszka Kozioł-Kozakowska, Paweł Matusik, Piotr Socha, Magdalena Olszanecka-Gilianowicz, Teresa Jackowska, Mieczysław Walczak, Jarosław Peregud-Pogorzelski, Elżbieta Tomiak, Małgorzata Wójcik

**Affiliations:** 1 Institute of Medical Sciences, Medical College of Rzeszow University, University of Rzeszów, 35-310 Rzeszów, Poland; 2 Department of Pediatrics, Faculty of Medical Sciences in Zabrze, Medical University of Silesia, 40-055 Zabrze, Poland; 3 Institute of Health Sciences, Medical College of Rzeszow University, University of Rzeszów, 35-310 Rzeszów, Poland; 4 Children’s University Hospital, Jagiellonian University Medical College, 31-008 Kraków, Poland; 5 Department of Pediatric and Adolescent Endocrinology, Chair of Pediatrics, Pediatric Institute, Jagiellonian University Medical College, 31-008 Kraków, Poland; 6 Department of Pediatric Nephrology and Hypertension, Chair of Pediatrics, Pediatric Institute, Jagiellonian University Medical College, 31-008 Kraków, Poland; 7 Department of Public Health and Social Medicine, Medical University of Gdańsk, 80-210 Gdańsk, Poland; 8 Chair and Department of Paediatrics, Gastroenterology, Allergology and Child Nutrition, Medical University of Gdańsk, 80-210 Gdańsk, Poland; 9 Department of Pediatrics, Gastroenterology and Nutrition, Jagiellonian University Medical College, 31-008 Kraków, Poland; 10 Department of Pediatrics, Pediatric Obesity and Metabolic Bone Diseases, Chair of Pediatrics and Pediatric Endocrinology, Faculty of Medical Sciences in Katowice, Medical University of Silesia, 40-055 Katowice, Poland; 11 The Children’s Memorial Health Institute, 04-736 Warsaw, Poland; 12 Health Promotion and Obesity Management Unit, Department of Pathophysiology, Medical Faculty in Katowice, Medical University of Silesia, 40-055 Katowice, Poland; 13 Department of Pediatrics, Centre of Postgraduate Medical Education, 01-813 Warsaw, Poland; 14 Department of Pediatrics, Endocrinology, Diabetology, Metabolic Disorders and Cardiology of the Developmental Age, Pomeranian Medical University, 70-204 Szczecin, Poland; 15 Department of Pediatrics, Pediatric Oncology and Immunology, Pomeranian Medical University, 70-204 Szczecin, Poland; 16 The College of Family Physicians in Poland, 00-209 Warszawa, Poland

**Keywords:** obesity, children, adolescents, join statement

## Abstract

Childhood obesity is one of the most important problems of public health. Searching was conducted by using PubMed/MEDLINE, Cochrane Library, Science Direct, MEDLINE, and EBSCO databases, from January 2022 to June 2022, for English language meta-analyses, systematic reviews, randomized clinical trials, and observational studies from all over the world. Five main topics were defined in a consensus join statement of the Polish Society of Pediatrics, Polish Society for Pediatric Obesity, Polish Society of Pediatric Endocrinology and Diabetes and Polish Association for the Study on Obesity: (1) definition, causes, consequences of obesity; (2) treatment of obesity; (3) obesity prevention; (4) the role of primary care in the prevention of obesity; (5) Recommendations for general practitioners, parents, teachers, and regional authorities. The statement outlines the role of diet, physical activity in the prevention and treatment of overweight and obesity, and gives appropriate recommendations for interventions by schools, parents, and primary health care. A multisite approach to weight control in children is recommended, taking into account the age, the severity of obesity, and the presence of obesity-related diseases. Combined interventions consisting of dietary modification, physical activity, behavioral therapy, and education are effective in improving metabolic and anthropometric indices. More actions are needed to strengthen the role of primary care in the effective prevention and treatment of obesity because a comprehensive, multi-component intervention appears to yield the best results.

## 1. Introduction

**Pediatric obesity is not a single nation problem, but it is one of the most important problems of public health** [1,2]. Although healthy eating patterns and regular physical activity (PA) help people achieve and maintain a healthy weight starting at an early age and continuing throughout life, every nation has unique cultural, economical, and health-care system conditions that make difficult to implement some detailed universal guidelines. Therefore, there is a need to publish local guidelines that will be in concordance with international, universal recommendations. This is the first position statement of the Polish Society of Pediatrics, Polish Society for Pediatric Obesity, Polish Society of Pediatric Endocrinology and Diabetes, and Polish Association for the Study on Obesity. The Expert Panel’s goal was to develop comprehensive evidence-based guidelines addressing to prevention, diagnosis and treatment of obesity and its complications in children and adolescents. The aim of the work was to assist pediatric care providers—pediatricians, family doctors, nurses, physiotherapist, registered dietitians, and psychologist in both the prevention and the identification and management of specific risk associated with obesity from infancy to adulthood.

## 2. Methods

Searching was conducted by using PubMed/MEDLINE, Cochrane Library, Science Direct, MEDLINE, and EBSCO databases, from January 2022 to June 2022, for English language meta-analyses, systematic reviews, randomized clinical trials, and observational studies from all over the world. The websites of scientific organizations, such as WHO, were also searched. Five main topics were defined: (1) definition, causes, consequences of obesity; (2) treatment of obesity; (3) obesity prevention; (4) the role of primary care in the prevention of obesity; (5) Recommendations for general practitioners, parents, teachers, and regional authorities.

## 3. Obesity—Definition, Causes, Consequences

### 3.1. Obesity—Definition

Obesity is a chronic recurrent disease related to excessive fat tissue accumulation that presents a risk to health. The diagnosis of overweight, obesity, and severe obesity is usually based on the measurement of high and weight, calculation of weight-to-length ratio in children below the age of 5 years and body mass index (BMI) in older children [3,4,5]. Indexes are assessed using child growth standards for age and sex. The advantages of these indexes are simplicity, low cost, universality of measurement, and assessment. However, it should be noted that they are not perfect in assessing the amount and distribution of fat tissue accumulation causing the development of obesity complications. In addition, they should be used with caution in a particular situation, for example, in athletes with high muscle mass or children with significant posture defects (scoliosis) related to the decrease of height measurement.

#### Diagnostic Tools and Data Interpretation

According to the World Health Organization (WHO), in children under the age of 5 years, overweight should be diagnosed if the weight-to-length ratio is greater than 2SD above the median of the child growth standard and obesity when this ratio is greater than 3SD above the median [3,5]. In children aged 3–18 years, Polish BMI percentile charts should be used, where overweight is defined as BMI above the 85th percentile (>1SD) and obesity above the 97th percentile (>2SD) [6]. WHO standards for children aged 5–19 years can be also used, with the overweight and obesity definition in accordance with Polish charts [7,8]. It is also possible to use older BMI percentiles charts for Polish children, published in 1999 by Palczewska and Niedzwiecka [9], where overweight is defined as BMI above the 90th percentile and obesity above the 97th percentile. However, using them, we risk underestimation of the prevalence of overweight compared to WHO charts.

Due to the high risk of metabolic and cardiovascular complications development, severe obesity should be specified. There are few definitions of severe obesity in children. We propose to use ONE, where severe obesity is diagnosed in children older than 5 years if BMI exceeds 3SD (99.9th centile) [5].

The accumulation of visceral fat tissue, which is an index of abdominal obesity related to a metabolic complication that can be used in children, is waist circumference [8]. It is measured at the level of the midpoint between the lowest rib and the iliac crest. For Polish children, centile charts for waist circumference for age and sex were developed within the OLA/OLAF project [10]. Up to the age of 16 years, waist circumference exceeding 90 percentile for age and sex defines abdominal obesity and is associated with increased cardiometabolic risk. In older adolescents, adult cut-off point values for abdominal obesity should be used (94 cm for men and 80 cm for females).

### 3.2. Specific Causes of Obesity

#### 3.2.1. ‘Simple’ Obesity

**The fundamental cause of obesity and overweight is an energy imbalance between calories consumed and calories expended** [11].

Weight status of children is closely associated with healthy lifestyle behaviors, such as physical activity, sedentary behavior, screen time, sleep, and dietary behaviors. Over 90% of obesity cases are idiopathic and less than 10% are associated with genetic and hormonal causes [12].

##### Unhealthy Diet

Poor eating habits, including inadequate intake of vegetables, fruit, and milk, and eating too many high-calorie snacks, play a main role in childhood obesity development. The body weight is regulated by various physiological mechanisms that maintain the balance between energy intake and energy expenditure. These regulatory systems under normal conditions, e.g., a positive energy balance of only 500 kJ (120 kcal) per day (approximately one serving of sugar-sweetened soft drink), would produce a 50 kg increase in body mass over 10 years [12]. Apart from excess caloric intake, very important for the development of childhood obesity are: incorrect, insufficient number of meals, skipping breakfast, drinking sugar-sweetened beverages, eating out, eating without hunger, and eating in front of the TV screen. In research conducted by Toschke et al. [13,14] on 477 children aged 5–7, the prevalence of obesity decreased with the higher number of meals consumed during the day. In the group of children who ate 3 or less meals per day, 15% of children were overweight and 4.2% were obese. Among children who ate 5 or more meals per day, the prevalence of overweight and obesity was 8.1% and 1.7%, respectively. People who regularly skipped breakfast had 4.5 times higher risk of obesity than those who regularly ate breakfast [15].

##### Sedentary Lifestyle

Research conducted in 49 countries in 2018 shows that 80% of Polish children lead a sedentary lifestyle. Our youngest took the penultimate place among their peers from Europe [16]. Children and adolescents spend between 246 and 387 min a day sitting [17]_._ European children spend up to 2.7 h watching TV a day [18]. Global trends, including excessive screen time spending, are creating a generation of ‘inactive children.’ During the pandemic, the percentage of children meeting the PA guidelines fell even further, while the percentage of children spending ≥ 2 h a day in front of a screen increased from 66% to 88% [19,20].

***Studies have shown that inactivity and sitting for more than four hours a day significantly increase the risk of cardiovascular disease, diabetes, and obesity, reduce sleep time, and also worsen prosocial and behavioral behaviors*** [21].

The latest reports about so-called obesity say that sedentary lifestyle and video games are the "new thrombophilia cocktail" in adolescents [22]. Weight gain is caused by more time sitting, but also by a greater consumption of snacks and sweets. Therefore, attention should be paid to activities that aim to modify a sedentary lifestyle in both school and home. Just three 5-min walks during the working day can reverse the changes caused by prolonged sitting in the peripheral arteries of the legs [23]. A 2017 study found that climbing stairs, considered high-intensity PA, burns more calories per minute than running [24].

Introducing active video games to increase daily energy expenditure in obese and sedentary children is not a substitute for sports activities but may contribute to increasing energy expenditure beyond the threshold of sedentary activity. Involving children in everyday activities, such as cleaning up after a meal, vacuuming, taking out the dog, throwing out garbage, reduces the time spent in a sitting position. Commercial breaks while watching TV may be used for this purpose.

A desk with an adjustable tabletop height or a seat in the form of a fitness ball will also force “active sitting”. Balls provide better concentration in learning than a short period of intense PA or lack of PA while studying [25].

The reduction in school sitting time and the use of active breaks in long sitting resulted in a significant improvement in the apoB/apoA-1 ratio with average effect sizes for TC, HDL-C, and TC to HDL-C ratio. The ability to concentrate attention is also improved. Measuring the number of steps and using health apps on your phone is an effective way to increase your child’s PA and thus weight loss [26,27].

Most studies use screen time as a replacement for total sedentary time. Media use does not represent all sedentary time [28]. Many interventions to reduce sitting time have focused on increasing PA. It has been shown that active children or athletes, compensating for their high PA, spend quite a lot of time on rest [29]. It is therefore important to correctly evaluate the sedentary time in children [30].

***The sedentary behaviors should be reduced in children with excessive weight to maximum 2 h per day***.

##### Sleep Restrictions

Sleep restriction in children and adolescents appears to be associated with an increased risk of weight gain, visceral obesity, and increased body fat mass, which may persist or manifest several years later. Increasing PA to at least 60 min per day promotes sleep hygiene and a reduced risk of overweight or obesity development [31,32,33,34,35].

**Excessive use of computer screens, tablets, smartphones, especially in the evening and at night may have a disruptive effect on sleep patterns, leading to a greater desire to eat at night and snack during the day** [36].

##### Psychological Mechanisms

**The psychological mechanisms behind the onset and maintenance of obesity are the object of inquiry in scientific studies for psychologists with different theoretical backgrounds** [37].

Excessive eating, the compulsive consumption of food, and affected somatic functioning (excessive body weight) are often signs of difficulties in a person’s psychological functioning. Obesity can be significant in terms of the mother–child relationship and other relationships in the family. A child’s obesity can play a role in experiencing emotions and in social relationships with peers and adults [37]. Additionally, some recent research points to a role of a chronic stress and alteration in glucocorticoids secretion and action in the development of overweight and obesity. Stress may play a major role in the development and maintenance of excessive body weight in individuals who have an increased glucocorticoid exposure or sensitivity due to increased long-term cortisol levels [38].

##### Binge Eating Disorder (BED)

Most of the excess eating that leads to obesity is not due to physical hunger but psychological causes. Certain cognitive schemas, therefore, trigger emotions and behavior towards food [39]. An important role in eating excessively is also played by ineffective mechanisms of emotional regulation related to the predominance of arousal processes over inhibition processes. This results in a unique style of coping with emotional tension, reduced ability to defer gratification, and impulsiveness [40].

***Binge Eating Disorder is characterized by the occurrence of recurrent, uncontrolled binge eating episodes, defined as eating significantly more food at a given time than most people would under similar circumstances and times*** [41].

The American psychiatric classification Diagnostic and Statistical Manual of Mental Disorders. Fifth Edition (DSM-5) distinguished BED as an independent disease entity, symbol 307.51 (F50.8) [42]. BED is now recognized as a separate type of eating disorder (in DSM-5) in addition to eating disorders such as bulimia nervosa (BN) and anorexia nervosa (AN) [43]. According to various data, this problem affects about 2–5% of the population and more often affects women [44,45]. This percentage increases significantly in obese people, ranging from 30% to even 36–42% [46], and 13–27% of obese individuals seeking treatment have ED [47,48]. To diagnose BED, ≥3 of the following indicators of control impairment for binge eating episodes must be present: eating until an unpleasant feeling of fullness appears; eating large amounts of food when not physically hungry; eating rapidly than usual; eating alone because of embarrassment; and feelings of disgust, guilt, or depression after an episode of binge eating [42]. Additionally, to diagnose BED, binge eating episodes must occur at least once a week for at least 3 months.

#### 3.2.2. Monogenic Obesity

***Monogenic obesity should be considered in children with early onset of weight gain (<2 years of age) and concomitant hyperphagia***.

Causes of secondary obesity include: genetic (monogenic, syndromic), endocrine, iatrogenic, or hypothalamic. Suspicion of secondary obesity should be assumed based on anamnesis (patient’s and family history) and physical examination with anthropometric evaluation, followed by additional diagnostics (differential diagnosis, hormonal, genetic, imaging assessment). The clinical features suggesting a genetic cause of obesity are: (1) history of consanguinity in the family; (2) intellectual impairment; (3) dysmorphic features; (4) organ/system specific abnormalities; (5) severe obesity of early development; (6) hyperphagia and food seeking behaviors; (7) other specific features/characteristic phenotypes. The confirmation of the diagnosis should be made on the basis of genetic testing.

Genetic obesity could be caused by a mutation in a single gene (monogenic), inherited recessively. It disrupts the regulatory system of satiety and hunger as well as energy expenditure. It is a rare condition and occurs in 3–10% of children with severe obesity. The most common gene mutation related to monogenic obesity is listened in Table 1. Personalized treatment is available for some mutations. Patients with leptin deficiency and biologically inactive leptin can be treated with recombinant human leptin (metreleptin) [49]. Melanocortin 4 receptor (MC4R) agonist, setmelanotide, is now approved for the treatment in patients with proopiomelanocortin, leptin receptor, and proprotei convertase subtilisin/kexin type 1 (PCSK1) deficiencies [50]. It is also known that patients with some mutations can be successfully treated with well-known drugs, e.g., glucagon-like peptide 1 (GLP-1) agonist, which is effective in weight reduction in patients with MC4R mutations, and obesity related to Kinase suppressor of Ras 2 mutation is well treated with metformin [51,52]. Identification of a monogenic background is also important in a patient’s qualification to bariatric surgery.

#### 3.2.3. Syndromic Obesity

***Syndromic obesity is usually related to dysmorphic features, mental retardation, and organ-system specific abnormalities***.

Syndromic obesity can be caused by a single gene mutation or a larger chromosomal region change that involves several/many genes. Despite obesity, it is usually related to dysmorphic features and characteristic of syndrome abnormalities. It is estimated that obesity could be a characteristic of almost 100 syndromes. The most common are Prader–Willi syndrome and Bardet–Biedl syndrome.

Prader–Willi syndrome is the most common form of syndromic obesity (1:15,000–25,000 births). It is caused by inactivation of the region 15q11-13 of the paternal chromosome. The characteristic features of this syndrome are: (1) severe neonatal hypotonia; (2) feeding problems and poor weight gain in the first year of life; (3) hyperphagia and obesity appear about 4–8 years; (4) characteristic dysmorphic features (small hands and feet, almond-shaped eyes, prominent nasal bridge, downturned lips, tall, narrow forehead); (5) hormonal deficiencies (growth hormone deficiency, hypogonadism, hypothyroidism); (6) intellectual impairment, speech difficulties, and behavioral disturbances. Genetic confirmation of the syndrome should be made as soon as possible in all neonates with hypotonia and in all older children with a characteristic phenotype. Implementation of recombinant growth hormone (rGH) treatment is possible in all children with Prader–Willi syndrome with a BMI below the 97th percentile. The therapy with rGH improves the body composition with the increase of lean body mass and decrease of visceral fat depot.

Bardet–Biedl syndrome is a ciliopathy caused by an autosomal recessive mutation in one of the 24 genes related to the function of the Bbsome—the protein complex involved in the function of the cillia. In addition to obesity, Bardet–Biedl syndrome is characterized by polydactyly, syndactyly, ataxia, hypertonia, speech difficulties, retinal dystrophy, intellectual impairment, renal dysfunction, and hypogonadism. Less common syndromes associated with obesity development, are for example: Alstrom syndrome, Borjeson–Forssman–Lehmann syndrome, Carpenter syndrome, CHOP syndrome.

#### 3.2.4. Obesity Associated with Endocrine Disorders

***Endocrine workup should be considered in any case of rapid weight gain with concomitant growth arrest/short stature***.

In the deferential diagnosis of obesity, some endocrine abnormalities (hypothyroidism, hypercortisolemia, growth hormone deficiency, pseudohypoparathyroidism) should be considered. In children with endocrine obesity, short stature, decreased growth velocity, and delayed bone age are typical (Table 2).

Iatrogenic obesity in children is related to chronic treatment with some drugs that affect appetite and metabolism (glucocorticoids, antiseizure drugs—valproic acid, atypical neuroleptics—e.g., clozapine, olanzapine, risperidone). Hypothalamic obesity arises from the disfunction of hunger and satiety centers in the hypothalamus and extreme hyperphagia. It could be caused by congenital abnormalities, head injuries, or a tumor located in the hypothalamic region [53,54,55].

### 3.3. Consequences and Complications of Obesity

#### 3.3.1. Arterial Hypertension

***Obesity is the main risk factor for arterial hypertension development in children and adolescents.***

Arterial hypertension (AH) is diagnosed in approximately 30% of pediatric patients with obesity, and the risk increases with the severity of the obesity [56,57,58]. Weight gain accounts for up to 75% of the risk of primary AH [59].

***Blood pressure measurement is recommended in all children with overweight or obesity.***

Early diagnosis of AH is crucial for any interventions that may reduce cardiovascular morbidity and mortality later in life [60]. Blood pressure (BP) should be measured in all children aged ≥3 years at least once a year and during any routine physician examination. However, in all children with overweight and obesity it is recommended to perform office BP measurements in all children with overweight and obesity <3 years of age, during routine health supervision visits, and visits related to health problems (at least once a year) [60,61,62]. It is also recommended to perform measurements in children <3 years of age if there are additional risk factors such as neonatal complications, cardiac malformations, genetic diseases, acquired or congenital kidney diseases, neoplasms, drug use, and diseases inducing increased intracranial pressure [60,62].

##### Office Blood Pressure Measurement 

The device used to measure BP must be validated for children with an appropriate size cuff to cover 80–100% of the individual’s arm circumference. Before BP measurement, we should ensure that the patient is sitting or relaxed for 3 to 5 min before. Measurement should be done three times with an interval of 3 min between measurements and use the average of the last two. The auscultatory method is recommended. If the oscillometric method is used, the device must be validated. If AH is detected by the oscillometric method, it needs to be confirmed using the auscultatory method. The diagnosis of AH requires a mean value of 3 independent measurements 95 percentile for age, sex, and height [60,61,62]. It is recommended to use the standards recommended by ESH (European Society Of Hypertension) (see online calculator: https://hyperchildnet.eu/blood-pressure-calculator/ accessed on 30 July 2022).

##### Home Blood Pressure Monitoring

Home BP in children with obesity correlates with target organ damage better than office BP, and may better reflect the effect of risk factors such as obesity and its metabolic complications [60]. It is recommended to perform home BP monitoring with a validated oscillometric device for 6 to 7 days, with duplicate morning and evening measurements [60,61,62].

##### Ambulatory Blood Pressure Monitoring

Ambulatory blood pressure monitoring is recommended in pediatric patients with severe obesity, with sleep-disordered breathing, any damage to the target organ (left ventricular hypertrophy and microalbuminuria) and normal office BP (suspicion of masked hypertension), type 2 diabetes mellitus, and chronic kidney disease [60].

##### Assessment of Potential Target Organ Damage in Patients with Obesity and AH

If arterial hypertension in a pediatric patient with obesity is confirmed, the following diagnostic tests are recommended: (1) assessment of kidney function: blood urea nitrogen, creatinine (and glomerular filtration by formula), electrolytes, urine examination, microalbuminuria; (2) evaluation of organ damage: echocardiography (to assess left ventricular hypertrophy or remodeling), fundoscopy.

##### Treatment of Arterial Hypertension in Children with Obesity

Non-pharmacological therapy, including both dietary modifications and PA, is of great importance in the management of AH in children with obesity. Daily high to moderate intensity exercise is recommended for 60 to 90 min. There are no contraindications to practicing certain types of PA. In the diet, particularly important are the limitation of sodium intake and the proper sodium to potassium ratio [63]. Pharmacological therapy should be considered in children with grade 1 hypertension in whom BP did not adequately decrease despite 6–12 months of non-pharmacological therapy and is indicated in children with grade 2 hypertension and/or target organ damage. The preferred drug classes are angiotensin-converting enzyme inhibitors (ACEIs), angiotensin II receptor blockers (ARBs), and dihydropyridine calcium antagonists [60,61,62]. If stage 2 hypertension, secondary causes, or any damage to the target organ are present, the patient must be referred to a specialist for further diagnostics tests and treatment.

#### 3.3.2. Prediabetes and Type 2 Diabetes Mellitus

***Assessment of glucose metabolism is recommended in all children and adolescents with overweight and obesity since the age of 6 years***.

Since there is some evidence that prediabetes is already present in approximately 5% obese children <10 years, it is recommended to measure fasting blood glucose in all children with overweight and obesity at the age of <6 years, as the first step to detect prediabetes and type 2 diabetes [64,65]. The screening must be repeated after 2–3 years, unless there is a rapid increase in weight or the development of other cardiometabolic complications [65]. The oral glucose tolerance test (OGTT) is recommended to be performed every two years in children with BMI > the 95th percentile > 10 years of age (or earlier, if puberty has already commenced) [66]. OGTT should be performed in a standard setting, with a glucose dose of 1.75 g/kg, a maximum of 75 g [67]. The use of glycated hemoglobin A1c (HbA1c) remains controversial in pediatric age because HbA1c has a lower sensitivity than fasting or OGTT plasma glucose [68].

***There is no recommendation to measure insulin concentrations during diagnostics obesity complications in children or adolescents***.

Fasting insulin concentrations show considerable overlap between insulin resistant and insulin sensitive youth. Therefore, there is no well-defined cut-off point to differentiate normal from abnormal and there is no universally accepted, clinically useful, numeric expression that defines insulin resistance [69,70]. We recommend against using insulin testing as a basis for making therapeutic decisions. Non-pharmacological therapy, including both dietary modifications and PA, is of great importance in the treatment of prediabetes in children with obesity. Certain medications (e.g., metformin, liraglutide) have potent effects on glucose levels, and their use may be considered under the supervision of an expert. Treatment options for pediatric patients with type 2 diabetes include insulin, metformin, and liraglutide (age limit according to summary of product characteristics) [66].

#### 3.3.3. Dyslipidemia

Dyslipidemias are disorders of lipoprotein metabolism that result in the following abnormalities: (1) high total cholesterol (TC); (2) high low-density lipoprotein cholesterol (LDL-C); (3) high non-high-density lipoprotein cholesterol (non-HDL-C); (4) high triglycerides (TG); (5) low HDL-C. Normal lipid and lipoprotein values in children vary by age and sex. In many patients, hyperlipidaemia is caused by some underlying "non-lipid" etiology rather than a primary disorder of lipoprotein metabolism. Among cardiovascular risk factors associated with increased morbidity and mortality, lipids and lipoproteins are of special importance, and in many studies, childhood obesity has been shown to be associated with increased levels of TC, LDL-C, and TG, and decreased HDL-C [71].

***The most frequent lipid disorders in children with obesityis combined dyslipidaemia characterized by moderate to severe elevation in TG and non–HDL cholesterol, decreased HDL-C, and mild to moderate elevation in TC andLDL-C*** [72].

Dyslipidemia is the most common consequence of childhood obesity and is present in as many as 43% of obese children. It is related significantly to insulin resistance as the latter is enhancing hepatic delivery of non-esterified free fatty acids for TG production and sequestration into triglyceride-rich lipoproteins. TGs are deposited in the vessel wall and initiate the process of LDL-C accumulation. They are strongly associated with the risk of developing atherosclerotic disease. LDL-C, very low-density lipoprotein (VLDL), and lipoprotein-a are the primary apolipoprotein-B containing lipoproteins implicated in the formation of atherosclerotic lesions. HDL-C has been thought to be protective through its ability to prevent oxidation of LDL-C [72].

Atherosclerosis starts at a young age, and the number of young people who develop atherosclerosis is increasing, especially children with risk factors such as familial hypercholesterolemia (FH), type 1 diabetes mellitus, and hypertension. In recent decades, hyperlipidaemia in children and adolescents has been increasing and many societies have identified these children as being at increased risk for premature atherosclerosis. The Bogalusa Heart Study demonstrated fatty streaks in 50% of cases between 2 and 15 years of age and in 85 percent of older subjects between 21 and 39 years of age [73]. The prevalence and extent of atherosclerosis found in the aorta and coronary arteries increased with increasing BMI, BP measurements, serum TC, and LDL-C. The degree of atherosclerotic changes increased with worsening severity and a greater number of risk factors [73].

***Assessment of lipid metabolism is recommended in all children and adolescents with overweight and obesity since the age of 2 years*** [74].

Screening of lipid levels in children reveals both genetic lipid abnormalities (e.g., including familial hypercholesterolemia, which affects 1 in 250 people), and dyslipidaemia, which responds favorably to lifestyle changes. In children with excessive weight, it is recommended to assess basic, fasting lipid profile (TC, TG, LDL-C, HDL-C) every 2 years. In children with any lipid disorders, the fasting lipid profile measurement should be repeated every six months to monitor treatment effectiveness.

In children with obesity, the diagnosis of dyslipidaemia requires additional lifestyle and lifestyle changes to reduce the risk and occurrence of cardiovascular complications. It is recommended primarily in the therapeutic management of dyslipidaemia caused by the obesity lifestyle change intervention. Even a slight weight loss is associated with a significant decrease in the TG concentration and an increase in HDL-C concentration. In addition to the recommended diet, it is helpful to achieve the desired effect, and therapeutic help is an adequate and regular increase in PA. As an adjuvant treatment use plant stanols, plant sterols, and ω−3 fatty acids are recommended. The plant stanols and sterol esters were shown to inhibit intestinal cholesterol absorption, leading to reduction in LDL-C up to 12%. Additionally mild reduction of TG during their usage is reported. Additionally, ω−3 fatty acids are widely accepted as a supplement used in children. Their exact mechanism of action is not clear, but they mainly reduce TG level. Usage of red yeast rice supplement, monacolin K, also known as lovastatin, an inhibitor of liver cholesterol synthesis, can be considered with caution. It is able to reduce LDL-C between 15–25% within 6–8 weeks of therapy. Due to its mechanism of action, similar to statins, the possible side effects should be closely monitored [75].

According to the National Institute of Health, Lung, and Blood (NHLBI) in cases in which non-pharmacological treatment has no effect, the use of pharmacological treatment should be considered. In accordance with the guidelines of the Polish Lipid Association (PoLA), after 6 months of low-lipid diet in children above the age of 6 with LDL-C concentration, persistent ≥190 mg/dL or ≥160 mg/dL and other risk factors, the statin treatment, together with non-pharmacological, should be considered [76].

#### 3.3.4. Digestive Tract Complications

***The most common digestive tract complication related to obesity in children is metabolic-associated fatty liver disease (MAFLD)*** [77].

MAFLD, previously called nonalcoholic fatty liver disease, may be present in 38% of overweight and obese children and adolescents [77]. The change in terminology aims to reflect the pathogenesis and risk factors for the disease, such as obesity [77]. It is a liver presentation of insulin resistance [77,78]. The risk of developing liver cirrhosis in children with MAFLD is much lower than in adults and amounts to 1–2% of children. In a child under the age of 10 years of age with hepatic steatosis, the secondary causes of the condition are common and should be considered (glycogen storage disease, hepatitis C virus, and others) [77].

In children with obesity, the diagnosis of MAFLD should be made on the basis of imaging and blood biomarkers. Liver biopsy is the standard of reference, although it is an invasive procedure and should be used only in rational cases. The most available and recommended imaging method for assessing liver steatosis is ultrasound. If available, magnetic resonance imaging (MRI) can be performed. Computed tomography (CT), although accurate, is not recommended due to the high X-ray exposition. The blood biomarker of MAFLD is an increase in alanine aminotransferase (AlAT) to more than twice the upper limit of normal. Unfortunately, both non-invasive methods (imaging and blood biomarkers) have moderate diagnostic accuracy. Additional evaluation of elastography could be useful, however, due to lack of validation, the accuracy remains uncertain [77,78,79]. From a clinical perspective, liver fibrosis is far more important than liver steatosis. Liver biopsy is a golden standard for assessment of fibrosis, but non-invasive methods in the future should replace liver biopsy once validated properly. Elastography, multiparametric RMI, and serum markers of fibrosis are investigated.

In the treatment of MAFLD, a diet with limited simple carbohydrates and increased fiber consumption is strongly recommended [79,80]. The introduction of supplements with ω−3 polyunsaturated fatty acids was postulated to reduce liver steatosis [80], but was not confirmed in other studies [81]. Pharmacological treatment of components of metabolic syndrome with metformin or statins should be considered once MAFLD is associated with lipid disturbances and insulin resistance. However, according to recent NASPGHAN guidelines, no pharmacotherapy is recommended [82].

Cholelithiasis in children is a rare disease with a prevalence of 0.13–0.22% and there is no indication for routine screening [83]. The main risk factors of cholelithiasis are elevated BMI and rapid weight loss. The risk of gallstones is higher in girls than in boys and increases with the severity of obesity and use of contraceptive pills [84]. In patients after sleeve gastrectomy, the incidence of symptomatic cholelithiasis is 3.5% over a period of 2 years [85]. Only in half of children is cholelithiasis symptomatic. In the diagnostic approach to cholelithiasis, abdominal ultrasound and liver enzyme assessment are crucial. Asymptomatic gallstones are diagnosed during a routine ultrasound examination. There is no evidence to routinely screen all obese children for cholelithiasis. However, abdominal ultrasound could be recommended in obese patients during/after rapid weight loss. In symptomatic patients (with pain in the upper right quadrant, vomiting, nausea, jaundice), the ultrasound is recommended.

Symptomatic cholelithiasis requires endoscopic cholecystectomy, and in asymptomatic patients, medical therapy with ursodeoxycholic acid (UDCA) can be considered under close observation [86]. UDCA treatment can also be effective in the prevention of gallstone formation in patients after sleeve gastrectomy.

***Obesity in children increases the risk of gastrointestinal reflux (GERD).***

GERD should be suspected if there is a characteristic clinical presentation (heartburn usually after eating, worse at night, chest pain, difficulty swallowing, regurgitation) [87]. GERD symptoms increased progressively with increasing BMI and waist circumference. 13.1% of obese children reported symptoms suggestive of GERD. Typical treatment and management is recommended, along with weight reduction to reduce symptoms.

#### 3.3.5. Polycystic Ovary Syndrome and Obesity Impact on Puberty

***In children with excessive weight, isolated, mild forms of precocious puberty (precocious pubarche, axillarche, thelarche) occur more often, and in obese girls, central puberty tends to start earlier*** [88].

The most common form of precocious puberty associated with obesity is precocious pubarche. It is related to insulin excess, which is often observed in obese children. Hyperinsulinemia can stimulate androgen production in the adrenals and ovaries. In prepubertal children, excessive adrenal androgen production can be clinically presented as precocious pubic and axillary hair occurrence before the age of 8 in girls and 9 in boys. It could be accompanied by pubertal sweat odor, mild acne, moderately accelerated growth, and bone age. It usually occurs more frequently in girls. In the hormonal assessment, isolated mild elevations of dehydroepiandrosterone sulfate (DHEAS) levels were observed. Less common in obese girls is isolated thelarche, as a consequence of androgen conversion to estrogens in the fat tissue. It is characterized by low concentrations of luteinizing hormone (LH) and estradiol with a mild increase in follicle stimulating hormone (FSH) levels. Height velocity and bone age are not accelerated. The mild forms of precocious puberty in obese children do not need any treatment. They are characterized by a stable course or very slow progression. In their treatment, serial observation and behavioral treatment of excessive weight were indicated.

***In girls with excessive weight, irregular menses occur twice more often than in non-obese peers***.

After menarche, obesity can be a cause of menstrual disturbances (heavy, painful menstruation, oligomenorrhea, secondary amenorrhea) and polycystic ovary syndrome (PCOS) [89,90]. PCOS in adolescent girls is characterized by menstrual irregularities and clinical hyperandrogenism and is associated with infertility, metabolic disturbances, type 2 diabetes, and cardiovascular disease in adulthood. In obese adolescents, it is related to hyperinsulinemia, which can stimulate ovarian and adrenal androgen production, as well as decrease the synthesis of sex hormone binding globulin (SHBG) in the liver, leading to excess androgen. According to the consensuses from 2017 and 2018 years, PCOS in an adolescent girls can be diagnosed if both criteria are met [91,92]:

(1) Menstrual disturbances (irregular menses, oligomenorrhea, and secondary amenorrhea). Irregular menses are defined as normal in the first gynecological year, however, the cycle duration of more than 90 days needs special attention. In the gynecological age of less than 3 years, the cycle is defined as irregular if it is shorter than 21 days and longer than 45 days. From the third gynecological age, the duration of the cycle should be between 21 and 35 days. Secondary amenorrhea is defined as a lack of menstruation for more than 3 months and primary amenorrhea as a lack of menarche at the age of 15 years or more than 3 years post-thelarche.

(2) Hyperandrogenism (clinical and/or biochemical). The clinical presentation of hyperandrogenism in adolescent girls is hirsutism, defined as excessive, coarse, terminal hair growth distributed in a male fashion, assessed by the Ferriman–Gallwey score for 8 or more points. It should be distinguished from hypertrichosis. Biochemical androgen access should be assessed on the basis of total testosterone and SHBG measurements and calculation of free/bioavailable testosterone or free androgen index.

The diagnosis of PCOS could be made if the gynecological age is older than 2 years and persistent menstrual disturbances are observed for more than 2 years. Other causes of menstrual disturbances and hyperandrogenism must be excluded (hypothyreosis, hypercortisolemia, hyperprolactinemia, congenital adrenal hyperplasia, androgen secreting tumor). The objectives of the treatment are regular menses and decrease in clinical features of hyperandrogenism. Despite a reduction in body weight, contraceptive therapy with antiandrogen action progestogen is indicated. In very young patients and in those with contraindications for estrogen therapy (venous thrombosis, migraine with aura), the natural progestogen therapy in the second phase of the cycle can be used. Antiandrogens (spironolactone, finasteride) are not registered in PCOS treatment, and their use should be considered with great caution. Metformin could be used in girls with PCOS and metabolic disturbances, and in addition to the improvement of metabolic profile, it could restore regular menses [91,92].

#### 3.3.6. Respiratory Disorders in Obesity

***In patients with obesity, the most commonly reported symptoms include an increased respiratory rate, dyspnea after low to moderate exertion, wheezing, and chest pain. Respiratory disorders such as bronchial asthma, obstructive sleep apnea (OSA) syndrome, or hypoventilation syndrome are more common in this group of patients*** [93,94,95]. Several review articles have appeared in recent years on the increased prevalence of asthma in obese patients. However, the topic remains highly controversial. Increased body fat may lead to systemic inflammation, increasing pro-inflammatory serum cytokines. With decreased lung compliance, lung volume, and peripheral airway diameter, bronchial hyperresponsiveness may also be important. A confirmatory factor for the effect of obesity on asthma is improved disease control when weight is reduced, as well as observed increased medication use and reported poor quality of life in obese patients compared to normal weight patients. The therapeutic efficacy of inhaled corticosteroids and their combination with long-acting beta agonists (LABAs) is significantly reduced. In spirometry, lower values of Forced expiratory volume (FEV1), total lung capacity (TLC), and functional residual capacity (FRC) are observed compared to patients with normal weight and bronchial asthma [94,96,97,98,99,100]. OSA is a condition manifested during sleep, characterized by repeated shallowing or complete absence of airflow through the upper airway with preserved chest and abdominal movements. It is associated with airflow limitation and consequent hypoxia (transient episodes of hypoxia and hypercapnia). It also causes sleep fragmentation through activation of the sympathetic nervous system and arousal. Its prevalence rate in children and adolescents with overweight or obesity ranges between 13–59% [101,102]. The features that raise suspicion of OSA include mouth breathing, pauses in breathing pattern, snoring during sleep, concentration problems, hyperactivity, headaches, and excessive daytime sleepiness. Untreated obstructive sleep apnea alters the quality of sleep and shortens the life expectancy of those affected [93,94,95,103,104]. Polysomnographic studies are performed to diagnose OBS. Weight loss is the first line therapy for obese children with OSA. For children with severe OSA, non-invasive ventilation (NIV) and continuous positive airway pressure (CPAP) can be the treatment of choice.

**Severe obesity** and OSA may lead to the **obesity**-**hypoventilation syndrome**, with hypoxia, hypercapnia, and reduced ventilatory drive. Hypoventilation syndrome occurs in severe obesity and its risk increases with increasing body weight. It is a chronic disease that reduces the activity of the patient in social life, reduces quality of life, and increases the risk of death. It is characterized by an increase in the partial pressure of CO_2_ and a decrease in O_2_ (PaCO_2_ > 45 mmHg and PaO_2_ < 70 mmHg), with other causes such as neuromuscular disorders, pulmonary vascular pathology, iatrogenic causes (drugs, psychoactive substances), metabolic diseases or respiratory and thoracic disorders excluded. Diagnostic criteria included a BMI ≥ 30 kg/m^2^ combined with hypoventilation PaCO_2_ > 45 mmHg (and during sleep > 55 mmHg for at least 10 min). Symptoms may initially be minor and as hypercapnia increases, headaches, impaired concentration, excessive sleepiness, confusion, and decreased exercise tolerance may occur [93,94,103,105,106].

#### 3.3.7. The Effects of Obesity on Musculoskeletal Health

Obesity is one of the most common conditions that negatively affects bone and joint health. Evidence showed positive associations between elevated body fat and the development of slipped capital femoral epiphysis [107,108], Blount’s disease, and genu varum [107,109,110]. Moreover, the risk of fracture, musculoskeletal pain [111,112], impaired mobility, and lower extremity malalignment are more common in children and adolescents with excess weight [110]. Persistence of obesity from childhood to adulthood may lead to an increased risk of osteoarthritis in the weightbearing joints, particularly at the knee [113]. Longitudinal studies indicated that increased body fat may influence the higher risk of incident and worsening joint pain [114].

#### 3.3.8. Renal Complications

***It is recommended to assess kidney function in children and adolescents with obesity. In adults, obesity is an independent risk factor for chronic kidney disease*** [69].

In children, it is not so obvious, but complications of obesity (e.g., arterial hypertension, dyslipidemia, insulin resistance, hyperglycemia, inflammatory state, and autonomous system dysfunction) can alter the kidney function [115]. Therefore, the basic evaluation of kidney function (creatinine level, glomerular filtration rate (eGFR [mL/min/1.73 m^2^] = 0.413 × body height [cm]/SCr [mg/dL]) [116] and urine analysis) should be performed in children with overweight and obesity. More detailed screening of kidney dysfunction (albuminuria, albumin/creatinine ratio) should be performed in patients with obesity and concomitant arterial hypertension and type 2 diabetes [60].

***The obesity seems to be an important risk factor associated with incontinence, but the interaction between these factors is complex and needs further investigation*** [117].

#### 3.3.9. Neurological Complications

***The obesity in children is a risk factor for migraine and idiopathic intracranial hypertension.***

Obesity in pubertal children is associated with a higher risk of idiopathic intracranial hypertension (pseudotumor cerebri) manifested with headache, nausea, vomiting, retroocular pain, and visual impairment [118,119]. However, the incidence of this condition is much less common in children than in adults. The possible pathogenesis of idiopathic intracranial hypertension in obesity is increased intraabdominal pressure, which in turn increases intrathoracic and intracerebral venous pressure. The most common clinical symptom of pseudotumor cerebri is headache, usually worse in the morning. It can be accompanied by nausea, vomiting, retroocular pain, decreased or blurred vision, diplopia, or even transient visual obscuration [118,119]. In 19% of children, it is associated with permanent visual impairment [120]. In younger children, irritability, apathy, and somnolence can occur. Less common are other nonspecific neurological symptoms—ataxia, dizziness, stiff neck, seizures, and facial nerve palsy. In some children, papilledema may be the only symptom of pseudotumor cerebri, without other symptoms. The diagnosis of idiopathic intracranial hypertension is the diagnosis of exclusion. Diagnostic criteria are the presence of characteristic clinical symptoms, including papilledema, in a patient with a normal level of consciousness, with normal neurologic physical examination (except cranial nerves), with normal findings on cerebrospinal fluid examination, neuroimaging studies, and documented increased intracranial pressure with lumbar puncture. Elevated intracranial pressure in a child with obesity can be diagnosed if the pressure of cerebrospinal fluid exceeds 28 cm H_2_O [121]. Magnetic resonance imaging shows signs of elevated intracranial pressure. Management usually covers medication: acetazolamide, which is a diuretic but also reduces cerebrospinal fluid production. Furosemide can be used together with acetazolamide or alone if the first medication is contraindicated. In some patients, the symptoms can resolve after the diagnostic lumbar puncture [118,119].

Obesity seems to be a risk factor for migraine progression and frequency of migraines. The prevalence of episodic migraine in obese children is higher compared to children of normal weight (8.9% vs. 2.5%) [122]. There is a relationship between headache physiopathology and the response of the central and peripheral mechanism to food consumption. The suggested mechanism includes obesity as a pro-inflammatory disease, which may be associated with neurovascular inflammation. Elevated levels of calcitonin gene-related peptides, dysregulation of the action of orexin, leptin, and adiponectin are possible proinflammatory factors related to obesity [123]. Therefore, weight control should be part of migraine treatment in a child with excessive weight.

#### 3.3.10. Mental Health Disorders

***Overweight and obesity can lead to physiological and biochemical disorders of the body, as well as a deterioration in self-esteem, well-being, and relations with the environment*** [124].

In children, they often initiate a negative emotional attitude towards themselves and a sense of non-acceptance by others. In the following years, they can lead to a feeling of rejection, loneliness, and obese teenagers very often feel disliked, lonely, and rejected by their peers. In such young people who are overweight or obese, there are noticeable difficulties in realizing their dreams, and excessive body weight makes it difficult for them to start their adult life and pursue their professional plans. Additionally, it favors an unattractive self-image, which may contribute to loneliness, a sense of regret, sadness, and even depression. Wardle et al. [125] found that body dissatisfaction was greater in obese children who developed it before the age of 16, therefore it should be identified as part of the multidisciplinary assessment. A referral to a specialist is needed in the suspicion of depressive and/or anxious symptoms, suicidal risk, dysmorphophobic traits, and eating disorders [42].

## 4. Treatment of Obesity

### 4.1. Weight Goal Reduction

***Weight loss goals are determined by the age of the child and the severity of obesity and related comorbidities***.

It has been suggested that in younger children with obesity the goal of treatment should be the stabilization of the body weight with successive BMI reduction. Maintenance of a stable weight for more than 1 year might be an appropriate goal for those children with overweight and mild obesity, because BMI will decrease as children gain height. In older children, weight loss is recommended to obtain the 85th percentile BMI. A weight loss of up to 1–2 kg/month is safe. Rapid weight loss is not recommended because of possible adverse effects on growth [65]. Bioelectrical impedance (BIA) is a useful method to assess the change in body composition in children [126,127].

### 4.2. Effectiveness of Nutritional Interventions

A stepwise approach to weight control in children is recommended, taking into account the child’s age, the severity of obesity, and the presence of obesity-related comorbidities [128,129]. Treatment of childhood obesity involves adherence to a structured weight reduction program individualized for each child, along with the adoption of a healthy diet and lifestyle. Anti-obesity medications play a limited role in childhood and are not recommended in younger children. Bariatric surgery is reserved for morbidly obese older adolescents, but its long-term safety data are limited in this age group [130]. The combination of increased PA and improved nutrition has shown promise as an intervention to combat obesity in children and adolescents [131].

### 4.3. Eating Behaviors and Lifestyle Modifications

***Obesity prevention and treatment should be a focus on diet, eating behaviors, and PA, and the reduction of body fat mass should be the summary effect of all this change***.

Efforts should be made to permanently change the lifestyle of the whole family [132,133]. Nutritional behaviors such as avoiding breakfast, irregular eating, snacking between meals, insufficient eating vegetables, and fruits are proven predictors of obesity development as well as sedentary lifestyle [134,135,136,137]. Special attention should be paid to them in patient education. The diet and other lifestyle modifications recommended for the treatment of obesity are summarized in Table 3.

### 4.4. Methods of Treatment by Dietary Modification

Dietary modifications are essential in the treatment of obesity, but there is a lack of one validated dietary strategy for weight loss in children. Various dietary modifications are used in scientific research for weight loss in children with obesity. As shown by these studies, diets with modified carbohydrate intake, such as low glycemic index and low carbohydrate diets, have been as effective as diets with standard macronutrients proportional to portion size control [138,139].

A well-balanced hypocaloric diet should be initiated among all obese children in consultation with a dietician [140]. The total daily energy of the diet should be calculated related to the ideal body weight for the height of the child and macronutrients proportion should fulfill the National Recommended Nutrient Intake Levels for Healthy Children (Table 4) [128]. The appropriate caloric restriction should be determined by a dietitian. The daily caloric value of the diet established to the ideal body weight for the height of the child may be reduced by 200–500 kcal. However, it should be noted that little to no evidence supports these specific recommendations. Rather, they represent an expert opinion. The reduced caloric intake should not be lower than 1000 kcal/day. For children with metabolic complications of obesity, especially insulin resistance and/or diabetes, more macronutrient modifications are needed.

### 4.5. Dietary Advice

In dietary treatment, decisions about the range of dietary restrictions must be made depending on the degree of excess weight and existing complications. Lifestyle recommendations listed in Table 3 are the basis of any intervention. Caution should be exercised regarding micronutrient and vitamin intake, particularly for the hypocaloric diet. If individually necessary, diet supplements should be used to meet the daily recommended intake [141].

### 4.6. Traffic Light and Modified Traffic Light Diet/Front-of-Pack (FOP) Nutrition Labeling

Food labels are considered a key component of strategies to prevention unhealthy diets and obesity. Nutrition labeling can be an effective approach to encourage consumers to choose healthier products. Interpretive labels, such as traffic light labels, can be more effective [142]. Appropriate labeling of foods with a Nutri Score can provide an important contribution to raising awareness for parents and children to support health-oriented purchases and influence improved diet quality [143]. Food is classified into one of three groups: RED, YELLOW, or GREEN. RED foods are foods that are high in fat and/or calories. This group also includes all sweets and sweetened beverages. GREEN foods are those that are low in fat and/or calories per serving. YELLOW foods fit between the two categories. Do not exceed 1200 to 1500 calories per day and do not eat more than four RED foods per week [144].

### 4.7. “Non-Restrictive” Approach

It does not consider the stated daily caloric intake or individual nutrients and focuses on eating foods that are low in fat and high in nutrients.

### 4.8. Industrial Diet (in the Original Replacement Meals, Replacement Meal)

Not recommended because efficacy and safety have not been tested in children/young adults.

### 4.9. Hypocaloric Diets with Low Glycemic Index

There was no evidence that the low glycemic index diet differed in effectiveness in reducing BMI or aspects of metabolic syndrome compared with other dietary recommendations in children and adolescents with obesity [138]. The low glycemic index diet was as effective as the low-fat diet. Studies do not indicate that a low glycemic index diet suppresses hunger or increases satiety in children and adolescents with obesity [145].

### 4.10. Physical Exercise

Eating habits and the level of PA affect human energy balance [146]. Current studies have already shown that, in childhood, there is an increase in the frequency of sedentary lifestyle, such as spending time on playing or working with a computer or watching television (TV) [147]. The increase in sedentary behavior and the reduction in the time spent in PA are important risk factors of the development of obesity in children [148,149].

Regular PA is associated with improvements in aerobic capacity, strength, muscle growth, bone mass, and body weight or body composition [150].

Metabolic benefits include lowering blood pressure, reduction of leptin, glycemia, and insulin resistance, improved lipid profile with lowering of TC, and increased HDL-C [151,152]. The physical activity reduces the levels of these inflammatory cytokines leading in addition to increasing anti-inflammatory cytokines, such as interleukin 10 and adiponectin, even without modifying diet or lifestyle changes [153,154,155]. Although exercise contributes to many health benefits, research suggests that exercise can play a role in both short- and long-term weight loss and maintenance. Obese children have to work harder than healthy weight children to perform the same task and therefore need an appropriate load. An exercise program for obese children should aim to increase caloric expenditure [156].

#### Modification of Physical Activity

The effects of PA may depend on the type of PA (aerobic exercise (AE), resistance training (RT), and mixed (CRAE)). For children with obesity, aerobic training (e.g., jumping rope, dancing, running, cycling) at moderate or moderate to vigorous intensity, for 30–60 min a day, 3–5 times a week is recommended [157,158,159,160]. Meta-analyses available in the literature suggest that AE interventions are effective in lowering fasting insulin levels, insulin resistance [161,162], and body fat percentage (BF%) [163], as well as improving blood lipid levels [164] in adolescents with obesity. In addition, AE training lowers overall body weight, BMI, and LDL-C [165].

RT increases muscle strength, power, and/or endurance and is usually done 1 to 3 times a week, while the number of repetitions, series, duration, and intensity of the exercises depend on the RT program. AE training is optimal for reducing BF%, while RT is optimal for increasing lean body mass [111].

Mixed training (CRAE) includes both AE and RT elements in a single exercise protocol to provide the benefits of each method is more beneficial for improvement of metabolic parameters and risk factors for cardiovascular disease than AE or RT alone. CRAE training generally involves performing a series of RT, one set of 8–20 repetitions of RT for the upper and lower body, followed by a series of AE, 20–30 min of moderate intensity, in one session of exercise. It has been shown that CRAE training improves both cardio-respiratory efficiency and muscle strength [160,166] and reduces the body fat, especially visceral [159].

***The most appropriate recipe for exercise to reduce obesity in children is the CRAE training protocol, which includes both muscle-toning (RT) and aerobic (AE) ingredients with an emphasis on fat reduction and long-term effects*** [167].

### 4.11. Family Cognitive Behavioral Therapy and Psychotherapy

***Psychological and/or psychotherapeutic support is an essential part of the treatment of obesity in children and adolescents.***

Isolated treatment of obesity is not effective due to its multifaceted nature and the multitude of factors that both condition and maintain it [168]. Adherence to medical treatment for obesity requires a wide variety of social and psychological skills. Psychological support aims to develop these skills to ensure compliance with medical recommendations [168].

***Psychological diagnosis can help with the correct choice of interaction methods and reduce the burden of care for the patient.***

At the beginning of the interaction, it is important to establish a proper psychological and/or psychiatric diagnosis [169,170]. Patients who will struggle with additional psychiatric disorders may require additional interventions before obesity treatment can be addressed [169]. A correct diagnosis is also intended to allow the most appropriate methods of interaction to be selected. Understanding the patient’s point of view can protect the medical team from burnout. This is because it allows for a realistic assessment of the pace and possibilities of the treatment process [171].

*Obesity is a chronic disease that triggers an adaptation process in the child. The adaptation process consists of different stages*.

As a chronic disease, obesity will provoke different responses in children and adolescents. At some stages of adaptation, it is possible that increased sadness and anger may occur. Being able to express these emotions and receiving help to experience them can contribute to better adaptation to chronic disease [172]. Healthy adaptation can, in turn, be associated with greater participation in the treatment process.

*Enhanced behavioral control is difficult in a dysregulated nervous system. Psychological support is intended to help restore balance to facilitate natural self-regulation in children and adolescents with obesity*.

When a child’s nervous system is balanced and when they are not overloaded with excess stress, they have greater access to specific cognitive skills and intentional actions [173]. A child or adolescent who is able to regulate his level of arousal is able to withstand discomfort more easily and cope with unpleasant emotions [172]. Psychological help for an obese child should be for healthy emotional regulation, as this will facilitate tasks that require self-control [172,173].

***Cognitive behavioral therapy is a recommended approach. This is because it allows the development of skills relevant to the perspective of lifestyle and behavior change.***

Cognitive behavioral therapy and its methods are recommended for the treatment of obesity [168,171]. An empathetic attitude on the part of the therapist is also considered important, which is expressed in not judging the difficulties experienced by the patient [174]. This is important because criticism does not serve the long-term achievement of goals and can lead to reduced motivation and poorer well-being [175,176].

Cognitive behavioral therapy is designed to help children master, among other techniques, (1) continuous monitoring of their behavior, (2) goal setting and management, (3) problem solving, (4) assertiveness, (5) ability to regulate emotions [168,171,177]. These skills are intended to help the child cope with temptations and maintain a healthy lifestyle. Additionally, cognitive interactions that change the thinking process from one that is maladaptive to one that serves health and life can be helpful [177].

The important role of motivation to maintain change should be considered [168]. If motivation is insufficient, the focus should be on the use of motivational dialogue [168,174]. Psychological support for children with obesity also has a protective function against psychological disturbances. Obesity is a risk factor for the development of psychosocial problems and mental disorders [170,178]. Children with obesity are more likely to be isolated from peers and treated as less attractive playmates [179]. This may cause the development of low self-esteem and as mood diseases such as anxiety and depression [170,178]. Psychological interventions can correct the psychosocial situation of children and allow for the restoration of healthy self-esteem. Psychotherapy is a necessary part of the treatment of eating disturbances such as emotional eating, BED, and night eating syndrome.

***Parental involvement in therapy is crucial for younger children.***

It should be remembered that for school children, parental involvement in the child’s therapy is important [177,180]. The influence of parents on children’s dietary compliance and PA is significant and important. The success of therapy will also depend on the functioning of the entire family system and the patient’s environment [181]. Therefore, systemic therapy may be a helpful solution in the treatment of childhood obesity [181].

### 4.12. Pharmacotherapy

***Pharmacotherapy for children or adolescents with obesity may only be considered after a formal program of intensive lifestyle modification has not been effective in limiting weight gain or improving obesity complications in adolescents aged ≥12 years with obesity defined as BMI corresponding ≥ 30 kg/m^2^ in adults***.

The only drug registered in Poland and Europe for people <18 years of age is the analog of the human glucagon—like peptide 1—liraglutide. While there are currently two formulations of liraglutide on the market, only one has been approved for the treatment of obesity under the name Saxneda. It may be used as a supplement to a healthy diet and increased PA [182]. Liraglutide, a glucagon-like peptide 1 (GLP-1) analogue, increases the postprandial insulin level in a glucose-dependent manner, reduces glucagon secretion, delays gastric emptying, and induces weight loss through reductions in appetite and energy intake [183]. Liraglutide under the name Saxenda approval was based on a 56-week, double-blind, randomized, placebo-controlled study in 251 pediatric pubertal patients aged 12 to 17 years. After a 12-week lifestyle run-in period, patients were randomized to Saxenda (3.0 mg) or placebo once a day. The mean change in BMI SDS from baseline to week 56 was −0.23 in the Saxenda group and −0.00 in the placebo group. The estimated treatment difference in the reduction in SDS in BMI from baseline between Saxenda vs. placebo was −0.22 (95% CI: −0.37, −0.08; *p* = 0.0022) [183].

Approved pharmacotherapy for obesity should be administered only with a concomitant lifestyle modification program of the highest intensity available and only by clinicians who are experienced in the use of drugs supporting the treatment of obesity and are aware of the potential for adverse reactions. Most adverse events of liraglutide are mild or moderate gastrointestinal events—including nausea, vomiting, and diarrhea [183]. The therapy should be discontinued and reevaluated if patients have not lost at least 4% of their BMI or BMI z-score after 12 weeks on the 3.0 mg/day or maximum tolerated dose [182].

It is not recommended to use metformin as a drug supporting the treatment of obesity in children and adolescents [184]. Metformin in children with overweight or obesity and metabolic complications reduces hepatic glucose production and increases peripheral insulin sensitivity [185]. It is not recommended to prescribe drugs supporting the weight loss off-label due to: (1) the limited data on safety and efficacy between children and adolescents, (2) the limited efficacy demonstrated in adults for most agents, (3) the need to weigh the relative risk of drug-induced adverse events in children and adolescents against the long-term theoretical potential of a drug to reduce obesity complications and mortality, and (4) the risk of creating a false belief that the drug can replace basic, effective, and safe methods of obesity treatment—change diet and increase PA [69].

### 4.13. Bariatric Surgery

#### 4.13.1. Requirements for Reference Centers

Bariatric surgery is more effective than conservative management [186]. Numerous studies have demonstrated the positive results of bariatric surgery on BMI reduction, reduction of blood pressure values, improvement in lipid and carbohydrate metabolism, and reduction of OSA [187,188,189,190].

***Bariatric surgery should only be performed in highly specialized centers based on the collaboration of an experienced multidisciplinary team capable of providing long-term care***.

The team should include a pediatric endocrinology and diabetes specialist or a pediatrician with experience in obesity treatment, a psychologist, an anesthetist, pediatric surgeon, a dietitian, and a physiotherapist. Depending on the needs, the team can be supplemented with specialists from other disciplines. The center should provide nephrology, gastroenterology, orthopedics, cardiology, pulmonology, psychiatric, and other consultations.

#### 4.13.2. Qualification

***Bariatric surgery should be considered in pediatric patients with BMI > 40 kg/m^2^ or BMI > 35 kg/m^2^ with associated: diabetes mellitus, prediabetes, hypertension, OSA syndrome, dyslipidemia (especially hypertriglyceridemia), signs of intracranial hypertension (pseudotumor cerebri), MAFLD, severe skeletal abnormalities, and urinary incontinence. An additional indication is a significant deterioration in patient quality of life and limitation of daily activities***.

The decision to qualify for treatment should be preceded by at least 12 months of treatment with modification of diet and PA and, in selected cases, pharmacotherapy. The best candidates for treatment are patients who have obtained satisfactory results from this treatment, but in spite of this, severity of obesity or obesity complications continue to threaten their health and life [65,191,192,193,194,195].

However, a prerequisite is that the patient and their parents are able to give their informed consent associated with a complete understanding of the nature of the surgery, the risks and benefits. It is also necessary to ensure that the minor patient has the support of his family during the preoperative and postoperative period. Consent should be preceded by psychological and psychiatric counseling for the patient and their family, and in selected cases by behavioral therapy. Currently, the prerequisite is no longer the sexual maturity of at least Tanner IV, the completion of the skeletal maturation, or the growth process, since no negative effects of bariatric surgery on growth and sexual maturation have been proven [196].

Contraindications to bariatric surgery include substance or alcohol addiction, pregnancy or planning a pregnancy within 2 years of surgery, breastfeeding, lack of informed consent and consent to surgery, lack of cooperation from the patient and family, untreated psychiatric illness, severe personality disorders, incurable debilitating illness that is life-threatening in the short term, and high risk of anesthesia for surgery. Relative contraindications to surgery or indications for its postponement are states of exacerbation or temporary imbalance of chronic diseases. With great caution, the decision about bariatric surgery should be made in patients with intellectual disability due to the problems with following the recommendation after surgery.

#### 4.13.3. Types of Bariatric Surgery

There are many types of bariatric surgery methods. Choosing the appropriate method is decided by the doctor in collaboration with the patient based on their health history, medical indications, and risk assessment. Laparoscopic surgery is the preferred surgical technique due to its lower surgical risk. Among the interventions with well-documented effects on weight reduction and expected metabolic outcomes, sleeve gastrectomy (SG) and Roux-en-Y gastric bypass (RYGB) are the most commonly performed in adolescents.

#### 4.13.4. Post-Treatment Monitoring

For at least two years after surgery, preferably until transfer to adult specialist care, the patient should remain under close multispecialty surveillance by the treating center.

Adolescents should have access to lifelong monitoring following bariatric surgery to ensure that nutritional requirements, and risks of developing post-bariatric surgery-related nutritional deficiencies, are monitored. The type and frequency of nutritional monitoring should reflect the bariatric procedure and may need to be individualized. The first post-operative visit should be done preferably after 7–14 days after the procedure. The next schedule of follow-up for the first 6 months includes 4 visits for 1, 2, 3, and 6 months. Until the second year after the procedure, subsequent visits should be carried out every 6 months. After 2 years, patients should be offered transition to adult care monitoring of nutritional status at least annually as a part of multidisciplinary-care management. Renal and liver function, full blood count, and ferritin have to be monitored at 3, 6, and 12 months in the first year and then at least annually. Regular monitoring of folates, vitamin B12, 25-hydroxyvitamin D, and calcium is essential. PTH levels have to be checked if not performed before surgery to exclude primary hypoparathyroidism. HbA1c and lipids have to be monitored in patients with preoperative diabetes and dyslipidemia. Requirements for other minerals and vitamins (zinc, selenium, thiamine, etc.) assessment are related to the specific symptoms and comorbidities [197]. Regular bone mineral density assessment (preferably annually) has to also be considered until peak bone mass has been reached [198].

Once the patient has reached adulthood, treatment should be provided in adult reference centers following bariatric surgery [191,192,196]. In the first-year post-operation, bariatric surgery results in a substantial weight loss of about 37%, leading to a significant decrease of all obesity-related metabolic complication, significantly improving health-related quality of life. However, in longer follow-ups, weight regain is observed in 50% of patients. Furthermore, reduced bone mass and nutritional deficiencies were reported in up to 90% of patients [199].

### 4.14. Effectiveness of Obesity Treatment in the Pediatric Population

Obesity treatment in the pediatric population aims to change the behavioral habits of patient and their closest environment (family, neighborhood, school) [200]. In long-term evaluation, those changes should result in improving the quality of liver and decreasing the risk of obesity complications [201]. However, in everyday practice, clinical evaluation, and study facilities, several anthropometric measurements should be used.

#### 4.14.1. BMI

The simplest, most often used measurement is BMI related to a standard population matrix—presented as standard deviation score (SDS), z-score, BMI centiles or 95th percentile for BMI (%BMIp95) [202]. These measurements are simple to use and repetitive. They can be performed in almost every facility with very limited equipment. Based on several measurements in time frames, it is easy to track any changes in the weight status of the patient using local or WHO based centile charts. The decrease in the SDS of 0.5 over 0–6 months of intervention is supposed to be associated with a decrease in body fat [203]. As is known, these methods have serious limitations. They do not really track changes in health status, only in relative body mass. Additionally, they do not track the decrease of fat tissue nor the increase in body muscles. This is why, nowadays, BMI-based measures can/should be used in population-based studies and screening procedures as the “best available” method. Unfortunately, there is no other golden standard for clinical practice. Waist circumference can be used as a measure of visceral fat change as it is more accurate for tracking changes in fat tissue, yet not effective in assessing increases in body lean or muscle mass [203,204].

#### 4.14.2. Other Anthropometric Measurements

More precise methods like bioimpedance, dual energy X-ray absorptiometry (DEXA), CT, or MRI are used mostly in tertiary reference centers for research purposes [205]. The availability of good quality and the reproducibility of bioimpedance is increasing, giving more accurate results on changes in fat and free fat mass. This method needs trained stuff and prepared patient—to give accurate and replicable measurements [206]. DEXA together with MRI are reserved mostly for clinical trials and have also some limitations—like the luck of standard charts/values for the pediatric population [205].

#### 4.14.3. Validation of Treatment Effects

There are limited data on the impact of body mass/fat mass reduction on long-term health effects—assessed from childhood until late adulthood [207]. The ones available are mainly observational or retrospective studies with limited factors accounted as possible bias. This also limits the usefulness of both anthropometric and equipment measures for assessing the changes of obesity [208]. Moreover, the assessment of changes in behavior is even harder, as it is mostly based on questionnaire/survey tools. Assessment of nutritional or PA habits has this important limitation of self-awareness and veracity [209]. PA is easily assessed by simple screening methods (step test, gait test, strength assessment) in both primary and reference centers. Therefore, the implementation of these methods would probably improve the quality of the assessment of changes in patients [210].

As of now, there is no ideal measure of the long-term effectiveness of lifestyle changes that can be used in a daily clinical practice. Long-term follow-up—30–40 years—to detect a reduction in obesity complications development and mortality is available in a limited number of population-based studies [208]. Moreover, focusing on weight and BMI-dependent measures may cause an increased risk of weight stigma and weight bias, which can contribute to discrimination, and can arise when children do not fit social norms for body weight or shape. This, in practice, can relay to the increased risk of depression, eating disorders, and low self-esteem, additionally contributing to overeating and decrease in PA behaviors [211].

All these factors contribute to the issue of qualitative and quantitative assessment and comparison of different public health, clinical and healthcare interventions. In most interventional studies, independently from their structure (family-based, school-based, individual, and group interventions), the BMI or related measure is still used as the most important and easiest in comparison measure. On the other hand, it is very hard to believe that there will be other easier-to-use measures, especially understanding the long-term consequences, relapse character, and multifactorial nature of obesity [212].

#### 4.14.4. Long-Term Monitoring

Monitoring and evaluation are an essential element of most processes, including the therapeutic process in obesity. The main goal of obesity treatment in children and adolescents is to prevent and treat obesity complications, including metabolic disorders, and to improve the quality of life of patients. Treatment of obesity in children should result in the development of health-promoting behaviors in the field of nutrition and PA, and their consolidation for the rest of the child’s life [213]. There is evidence of short-term efficacy of multi-module interventions in the treatment of childhood obesity for age groups up to 6 years [214], 6 to 11 years old [214], and from 12 to 17 years of age [202].

Obesity as a chronic disease requires long-term lifestyle changes and thus long-term patient monitoring [215]. One should remember about the possibility of recurrence of the disease, and thus the reevaluation of the causes of its occurrence and the selection of appropriate treatment methods, tailored to the patient’s abilities and needs. There are no long-term patterns of how often an obese patient should undergo specialist visits when he or she obtains the goals set in the treatment plan—not only weight reduction, but also all above behavior modification. The regular visits at intervals that would allow the therapeutic effect to be maintained and early identification of body weight gain should be recommended. In the case of bariatric surgery, except for the first two years after surgery, one visit per year is recommended in the following years [216].

## 5. Obesity Prevention

***According to the recommendations of the WHO, prophylactic/preventive activities should occupy the leading position among activities aimed at reducing the occurrence of overweight and obesity in the population of children and adolescents*** [217,218,219].

Increasing incidence of overweight and obesity in children and adolescents in European countries requires quick and effective measures taken by governmental and non-governmental institutions, local governments, the food industry, health care system, educational institutions, and individually at the level of families and individuals themselves.

### 5.1. The Importance of the Family

Parenthood is based on caring for children and their development. It has been confirmed that the basic criterion for the proper development of adults as parents is the successful development of their offspring [220]. Parents’ knowledge and participation is crucial for them to take appropriate, necessary actions to maintain their child’s health.

The guidelines emphasize that all activities aimed at preventing excessive weight gain, improving diet, and the level of PA in children and adolescents must actively involve parents and guardians. Education aimed at parents should emphasize the importance of their role in modeling health behavior (diet, exercise), control, support, and motivation.

In the early stages of life, the mechanism of learning through imitation, i.e., repeating, recreating activities, behaviors, and choices of parents, plays a special role. It is important that parents and guardians verify their behavior. Data show that most adults’ behaviors are motivated by experiences from their own family home [221]. Educational activities are also necessary regarding the principles of proper nutrition of children at various stages of a child’s life, the recommended time and forms of PA, and the impact of excessive body weight on the child’s health and the reduction of obesity. The key is for parents to understand the essence of the disease, which is obesity—parents often do not perceive the excess body weight of a child in terms of a disease and thus do not take any intervention measures [222,223].

The role of the parent in the prevention of obesity should focus on shaping the correct nutritional behavior, starting with the proper nutrition of the parents themselves, exclusive breastfeeding of infants up to the age of 6 months, in accordance with the recommendations of expanding the child’s diet, and then maintaining a proper diet. Parental control of the child’s menu is also important when the child begins to make food choices on its own. The parent’s task is to take dietary modification measures to prevent excess weight gain and, if necessary, reduce excess body weight.

The correct formation of patterns of behavior related to PA is also the responsibility of parents and guardians who, by their own example, stimulate children to engage in PA. The parent’s task is to allow the child to comply with the WHO guidelines on PA: children and adolescents require, on average, 60 min a day of moderate to high aerobic intensity [224].

In conclusion, the role of the parent and the family is to create an environment that models appropriate health behavior, flexible, and ready to change in the event of a threat to the health of the child, but also supports the child in pro-health behaviors. These family tasks should be carried out in the home environment but also outside it, involving other key people in the process (e.g., grandparents, neighbors, friends, helping with caring for the child). Lifestyle modification in child is most effective when changes affect all family members. Behavioral correction may also bring health benefits to households with healthy body weight, while not causing a feeling of exclusion or stigmatization in a child with excessive body weight [225].

### 5.2. Prevention–Prenatal Period

***The prevention of childhood obesity should be started at the pre-pregnancy period because both preconception and perinatal maternal health, and especially BMI, consistently predict excessive weight in the offspring*** [226]. The modifiable risk factors for childhood overweight and obesity development related to pregnancy are: high maternal preconception BMI, excessive weight gain during pregnancy, maternal gestational diabetes mellitus, hypertension, and smoking during pregnancy [227]. These factors are related to newborn’s low birth weight, macrosomia, and also to small-for-gestational age (SGA) and large-for-gestational age (LGA), which are related with increased risk of high fat mass and metabolic disturbances in later life [227]. It was shown that women who have excessive weight were twice as likely to have an overweight or obese child compared to women with normal weight [226]. In addition, the disturbed intrauterine environment caused by an elevated glucose level in the mother’s blood is related to an increased risk of increased birth weight, obesity, and metabolic disorders later in life [228]. Other prenatal conditions, hypertension, and smoking during pregnancy, are associated with the risk of low birth weight and SGA.

Prevention actions should focus on modifiable pregnancy-related risk factors for childhood overweight and obesity. Healthy lifestyle, PA, and balanced diet leading to maintain the normal body weight before conception, as well as proper weight gain during pregnancy, should reduce overweight and obesity risks in a child [229]. In women at risk of gestational diabetes mellitus, the prevention, early diagnosis, and proper treatment of glucose metabolism disturbances are essential for child’s health [228]. In addition to monitoring the glucose level and possible insulin treatment, a diet with decreased carbohydrates and PA are crucial. In pregnant women with low weight and undernutrition, the risk of having infants with SGA or low birth weight is high. For mothers, an energy-balanced, protein supplemented diet could be considered [227].

### 5.3. Nutrition for Children 0–2 Years

Proper nutrition in the first period of life is primarily to meet the demand for energy and necessary nutrients, ensuring proper physical and psychomotor development. This will help prevent overweight development. It is also recommended to avoid excessive weight gain and/or an increased weight-to-length ratio from the first months of life. Children with obesity are more likely to become adults with obesity [230].

The goal to be pursued is exclusive breastfeeding for the first 6 months of life. Partial or shorter breastfeeding is also beneficial. Breastfeeding should continue for as long as desired by the mother and baby [231]. Human food produced in sufficient quantity fully satisfies the infant’s need for all necessary nutrients, while ensuring its proper development in the first half of its life. Healthy infants 1–6 months of pure breast feeding consume approximately 75 ± 12.6 g of milk from one breast and 101 ± 15.6 g from both breasts.

The average number of feedings decreases with the age of the baby and is as follows:

▪ in the first six months of life, 8–12/24 h
▪ in the second half of the first year of life, 6–8/24 h
▪ in the 2nd year of life, 3–6/24 h [232].

It should be aimed to ensure that a child after 1 year of age was no longer fed at night. Infants not fed naturally should receive breast milk substitutes. Based on the consensus of experts, a recommendation was formulated that after reaching the 12th month of life, breastfeeding should continue for as long as desired by the mother and baby. During this time, it is recommended to provide complementary foods. The introduction of complementary products should start when the infant shows the developmental skills needed to consume them, usually not earlier than 17 weeks of age (beginning of the fifth month of life) and not later than 26 weeks of age (beginning of the seventh month of life) [231].

In the nutrition of toddlers, there are significant changes in the eating patterns related to the transition from a typical milk (liquid) diet to a more varied diet (infant diet → transitional diet → family, table diet). During this period, behavior and food preferences also form. The demand for energy and most nutrients in toddlers is reduced per 1 kg of body weight compared to infancy, and for some components it remains relatively constant [233].

### 5.4. Nutrition from Preschool to Adolescence

Eating a variety of vegetables and fruits, whole grains, a variety of lean protein foods, and low-fat and fat-free dairy products is essential for maintaining a normal body weight and health [234]. It is also recommended to limit foods and beverages with added sugars, solid fats, or sodium, as well as alcoholic and energy drinks. Rational nutrition should optimally include five meals a day. The appropriate proportions between meals and the regular hours of their consumption should be promoted [235,236].

#### 5.4.1. Physical Activity

The first years of life are essential for starting obesity prevention focused on promoting and maintaining an appropriate level of PA. Prevention strategies should include families, schools, social networks, media, and the general community, which should promote a healthy lifestyle by giving an example to follow or providing a supportive environment [237]. For many children, maintaining an appropriate level of PA may be sufficient to prevent obesity. Children who are physically active have lower body fat content than their physically inactive peers [238]. The 2020 PHYSICAL activity guidelines call for children and adolescents aged from 5 to 17 to accumulate at least an average of 60 minutes of moderate- to vigorous PA (MVPA) per day, mostly aerobic. They also recommend that vigorous physical activities and exercise to strengthen muscles and bones be undertaken at least 3 days a week. Infants (<1 year) should be encouraged to be physically active several times a day by supervised, interactive floor-based play. Toddlers (1–2 years) and preschoolers (3–4 years) should accumulate at least 180 min of PA at any intensity, including MVPA, spread throughout the day. A higher level of PA than the recommended minimum is associated with additional health benefits, such as increased physical fitness (cardiorespiratory and muscular fitness), decreasing of body fat, improvement of cardiometabolic health (BP, dyslipidaemia, glucose, and insulin resistance), improvement of bone health, cognitive outcomes, and mental health [239,240].

#### 5.4.2. Sedentary Behaviors

There is evidence to suggest that in the pediatric population, greater time spent in sedentary behavior (especially screen time, including TV viewing) is associated with excessive body weight and poorer health outcomes, such as decreased physical fitness and cardiometabolic health [241]. This can be explained by the fact that the screen time competes with PA time, and therefore displaces energy expenditure [242]. Moreover, screen time is often associated with increased consumption of food, exposure to high-calorie, nutrient-poor food, and shorter sleep duration [243]. Reallocation of sedentary time to MVPA is related with a reduction of adiposity among youth [244]. Evidence suggested that screen time over 2 h per day was related to a higher risk of overweight/obesity in children [245] Therefore, it is recommended to limit the time spent in sedentary behavior to 2 h per day by breaking up long periods of sitting as often as possible. Less time spent in sedentary behaviors seems to have better health outcomes [246,247]. The 2020 WHO guidelines call for children and adolescents to limit sedentary behavior, especially the amount of time spent on recreational screen time [135].

#### 5.4.3. Sleep—Preventive Behavior—Sleep in Obesity

As part of the prevention of the development of obesity, the time spent watching TV, playing computer games, and using mobile phones should be limited. The time spent in older children (i.e., >2 years of age) is up to a maximum of 2 h per day completed at least 30 min before going to bed. In infants and children up to 2 years of age, complete use of multimedia devices is discouraged. These behaviors can have a disruptive effect on sleep patterns, leading to a greater desire to eat at night and snack during the day. Short duration of sleep is a potential risk factor for obesity because it affects the neuroendocrine and metabolic systems. Sleep restriction in children and adolescents appears to be associated with an increased risk of weight gain, visceral obesity, and increased body fat mass, which may persist or manifest several years later. Increasing PA to at least 60 min per day promotes sleep hygiene and a reduced risk of overweight or obesity development [31,32,33,34,35].

#### 5.4.4. Role Involving the School Community

The school environment, after the home environment, is the second most important center where the lives of children and young people are concentrated. A child suffering from obesity usually stands out in their peer group: they are larger, often less physically fit. In the social aspect: children and adolescents with excess body weight face nonacceptance or even rejection from their peer group. The effect of this is lowered self-esteem, which becomes a common problem in the mental sphere, leading to the development of depression, behavioral disorders, and a reduction in quality of life [248]. A child with excess body weight in the school environment can become a victim of verbal, physical, and mental aggression, stigmatized due to obesity.

The school environment, which is the place of contact between the child and the peer group, may positively or negatively influence the shaping of social relationships. A well-prepared school environment, educated teaching staff in the field of obesity, including stigmatization, can effectively support the building of positive behaviors and attitudes toward a child suffering from obesity. A properly moderated peer group can be a support group for a child with overweight and obesity, strengthening his self-esteem and positive self-image. Acceptance of the peer group reduces the feeling of fear and guilt often accompanying children with overweight and obesity, which significantly interferes with the process of adaptation to the environment [249].

***From the perspective of the organization of the school environment, the preventive programs in which the school participates are important, focusing its activities on the area of healthy eating, PA, or directly prevention of obesity development.***

Active participation in this type of initiative increases the chances of a child with overweight and obesity to return to a normal body weight by shaping appropriate prohealth behaviors. In addition, an important aspect is also the organization of the school nutrition system: the principles of the school cafeteria (quality of meals served, portion sizes, and hours of serving meals), the school shop (quality of the available assortment), the presence of vending machines (the quality of the assortment available in them), and finally the organization of breaks between lessons to allow children to eat a meal in peace. The way of organizing PA in the school as part of physical education (PE) lessons is also important, as well as extracurricular activities. It should be noted here that the correct planning of PE classes in the hour grid may be a factor that increases the active participation of children and adolescents in these classes. The method of conducting PE classes, which should be a form of fun, is also important. Sports rivalry and discriminatory situations should be avoided.

It should also be mentioned the role of the school nurse, who, in the pre-school and school period, as part of primary health care, together with a pediatrician or family doctor, provides preventive care for children. Balance examinations are an opportunity to assess the health of children, monitor their development, diagnose irregularities, and take corrective actions to detect deficits. The school nurse, by being present in the school environment, can stimulate actions aimed at improving diet and PA [225]. Nevertheless, it should be noted that the field of activities of the school nurse, the scope of duties, and the proper use of the obtained data require improvement [250].

### 5.5. The Social Environmental Factor in the Prevention of Childhood Obesity

#### 5.5.1. The Influence of Culture on Childhood Obesity

Culture is believed to significantly affect children’s body weight. First, the development of body image occurs in a cultural context and differs in shared understandings as to valued and disvalued body image [251,252]. In some communities, thinness is considered beauty, while in others, a plump child is considered healthier [253]. Parents’ perceptions of their children’s body mass varied geographically. Parents from Southern Europe more often misclassified overweight children as normal weight compared with parents from Central and Northern Europe [254]. Moreover, cultural factors have a strong influence on eating habits and behavior and, consequently, the body weight of children and adolescents [255,256]. Eating traditional foods with the family may be associated with lowering the risk of obesity in some children (e.g., Asians) [257] or increasing the risk of obesity in other children (e.g., African Americans) [258]. Culture also influences the preferences and possibilities of practicing physical activity. Children model the types of physical activity undertaken by parents. Therefore, in a culture that views rest after a long working day as healthier than physical activity, a parent is less likely to have children who understand the importance of exercise for health and well-being [259].

#### 5.5.2. The Influence of Policy on Childhood Obesity

The progressive phenomenon of overweight and obesity in children and adolescents requires action by governmental organizations. In Poland, the National Health Program 2016–2020 was developed where obesity was recognized as a disease of civilization and its treatment as one of the priorities [260]. The previous National Program for the Prevention of Overweight and Obesity and Chronic Non-Communicable Diseases through Improved Nutrition and Physical Activity for 2007–2011 was based on increasing public awareness of the importance of adequate nutrition and physical activity in relation to health maintenance [261]. In 2015, a law was introduced concerning groups of foodstuffs for sale to children and adolescents in units of the educational system and the requirements to be met by foodstuffs used in the collective nutrition of children and adolescents in these units. It prohibits the sale of unhealthy food products in school canteens [262].

#### 5.5.3. National Level Approach and Childhood Obesity

The main reasons for the development of childhood obesity are insufficient physical activity, improper nutrition of children at home, resulting from the lack of knowledge of parents, acquiring knowledge about children’s nutrition mainly from the Internet, and easy availability of unhealthy food for children.

The World Health Organization points out that only an integrated effort can help to be successful in raising awareness and changing health behaviors in order to prevent the trend of an increase in the prevalence of obesity in children [263]. Educational activities aimed at changing lifestyles are of particular importance in the prevention programs for obesity in children and adolescents. She draws attention to the importance of proper care for a pregnant woman, breastfeeding, and recommends the introduction of taxation of sweetened drinks and the inclusion of obesity prevention in the tasks of the school nurse [264].

In recent years, several initiatives have been taken in Poland to tackle the problem of child obesity. This problem was included as a priority and included in the National Health Program. The National Program for the Prevention of Overweight and Obesity and Chronic Non-Communicable Diseases by improving nutrition and physical activity for 2007–2011 was developed. It was based on increasing public awareness of the importance of adequate nutrition and exercise in relation to health.

In 2015, an act was introduced on groups of foodstuffs intended for sale to children and adolescents in education system units and the requirements to be met by foodstuffs used as part of mass nutrition of children and adolescents in these units, the so-called “Shop act”. It prohibits the sale of unhealthy food products in school shops [262].

A 21-day program of physical activity and clinical dietetics for obese children aged 15–17 was also introduced to examine the lipid profile and glutathione levels, and the obligation to record a conversation about children’s nutrition in the Children’s Health Book [265].

“Bicycle May” is the largest campaign in Poland promoting an active way to school, a healthy lifestyle and sustainable mobility among preschool children, primary school students, teachers, parents, and guardians. Bicycle May, through fun combined with elements of competition, popularizes the bicycle as a means of transport to school, teaching good and healthy habits that persist even after the end of the campaign. There are prizes for the most active participants, classes, and institutions [266].

This is one of the elements of creating a healthy environment in kindergartens and schools, but is also for the employees of institutions and parents of students.

As part of educational programs addressed to students, “Fruit and vegetables at school” and “Milk at school” were introduced. Their goal was to improve the eating habits of schoolchildren by promoting and increasing the consumption of vegetables, fruit, milk, and dairy products, i.e., products important for the proper development of a child, and at the same time often deficient in the daily diet. Since 2017, both programs have been combined into the “Program for Schools”, which currently covers students from grades 1–5 of most Polish primary schools [267].

In Poland, legal regulations on advertising food in programs for children were included in the Broadcasting Act of 29 December 1992, as amended (2015), according to which "programs for children should not be accompanied by commercial communications regarding food or beverages containing ingredients, the presence of which in excessive amounts in the daily diet is not recommended” [268].

In 2018, the educational program “5 portions of health at school” began. Its aim is to draw attention to the need for education in the field of proper nutrition from an early age and at the same time to start it early. The program is addressed to students of 2nd and 3rd grades of primary schools from all over Poland and their teachers, school principals and school nutritionists. 2016–2020 [269].

The “Keep Fit” program, co-implemented by the Chief Sanitary Inspectorate and the Polish Federation of Food Producers, is an initiative promoting a healthy lifestyle, combining balanced nutrition with regular physical activity [270].

Although obesity was recognized as a civilization disease in the National Health Program for 2016–2020, and its treatment as one of the priorities, the report of the NIK (Najwyższa Izba Kontroli Supreme Audit) shows that the actions taken by successive health ministers not only did not lead to a decline, but even to inhibiting the growth rate of the number of children and adolescents with excess body weight. The scale of the problem was growing, and the effectiveness of the therapy was modest. In the opinion of the Chamber, the reasons for such low effectiveness of treatment were diagnostic errors and outdated methods of therapy, but most of all the lack of access to treatment, mainly due to the lack of specialists [271].

### 5.6. The Role of Primary Care in the Prevention of Obesity Development and Its Treatment in Children

Epidemiological data show that a family doctor encounters obesity and its complications daily in their practice. The employees of primary care facilities are the basic representatives of medical care to have first contact with patients who suffer from obesity. Treatment of overweight and obesity prevents the development of complications. Sometimes, it also allows to heal already developed complications. Consequently, the main task of primary healthcare workers—family physicians, pediatricians, and nurses—is the diagnosis and treatment of overweight and obesity. As shown in previously published studies, parents often do not perceive the overweight and obesity of a child in terms of a health problem and thus do not take any intervention measures [222,223]. Professional medical support provided to overweight and obese patients benefits both the patient and their family, as well as the whole society, reducing the direct and indirect costs of obesity.

***Primary health care workers have the greatest opportunity to observe changes in body weight in their patients and to identify environmental determinants and psychological factors that contribute to the emergence and perpetuation of abnormal behavior. At the local level, they are responsible for promoting the health of their charges.***

Patients who choose a primary care physician remain under their care for many years. This allows the relationship to be consolidated, trust built, and thus continuously monitor and motivate patients to pro-health behavior. The long-term relationship with the patient, the knowledge of their medical history, and frequent contact are of particular importance in the case of diseases that develop over many years and chronic diseases such as obesity. Monitoring development, including changes in body weight in a child, makes it possible to capture the growing excess body weight at an early stage and initiate therapeutic measures as soon as possible.

However, the diagnosis and treatment of obesity is one of the main problems in not only primary, but also specialist health care in Poland. More actions are needed to strengthen the role of primary care in the effective prevention and treatment of obesity.

## 6. Recommendations

*Recommendation for general practitioners (GPs)*

Primary care physicians who provide preventive care to children should:

- as part of each contact with the child, especially with vaccinations and periodical check-ups assess their nutritional status and if BMI indicates excess body weight, in particular obesity, which is a chronic disease, they should make such a diagnosis and undertake appropriate treatment;
- educate the patient’s parents and the child about a healthy lifestyle based on their own observations and an interview on the diet and level of PA;
- educate parents about the dangers of obesity and its complications;
- cooperating with representatives of other medical professions (dieticians, physiotherapists, psychologists) to improve the effectiveness of caring for a child with obesity;
- inform parents of children with overweight and obesity about available support methods and places where such support can be obtained operating in the region (information about specialist clinics, available preventive programs and health policy programs);
- cooperate with local government authorities to build an effective system of support for patients with excessive weight in the region.

*Recommendations for Parents*

Parents of a child who is overweight or obese play a special role in the prevention of obesity, and their correct attitude directly translates into the effectiveness of the therapeutic process. Top recommendations for parents include:

- building appropriate health behaviors from the earliest stage of a child’s development, through own appropriate behavior, modeling the child’s behavior;
- exclusive breastfeeding of infants up to 6 months of age and expanding the menu of young children according to applicable recommendations;
- organization of the diet based on the principles of healthy eating;
- enabling the child to carry out min. 60 min of PA, according to the guidelines of the WHO;
- if necessary, modification of the lifestyle of the whole family: diet and exercise to stop the accumulation of overweight and obesity and achieve a reduction in excess;
- ensuring that sleep time is appropriate for the age of the child;
- active participation in preventive health care;
- create an environment that supports the child’s pro-health behavior.

*Recommendations for teachers*

The teacher, who is the guardian of the child in the school environment, is obliged to ensure their safety and support their physical, mental, and social development. Top recommendations for teachers include:

- support overweight and obese children in a peer group—building a child’s self-esteem, including, in particular, a child with overweight and obesity—showing interest, expressing recognition, and appreciation of the child. Such activities may have a protective effect, on the one hand, preventing the development of lowered self-esteem in a child and, on the other hand, preventing stigmatization in the peer group;
- influence the social position of a child with overweight and obesity in the peer group, e.g., by showing the group of strengths of individual students, build their position by counteracting exclusion;
- undertake activities that will support the return to normal body weight, e.g., by establishing group rules for bringing sweet products to school;
- motivate the child and support the reduction of excess body weight;
- shape, by their own example, the positive behavior of your pupils, for example, through the right food choices, not rewarding children with sweets, and a reasonable choice of places where children eat meals during school trips;
- enabling students to take an active part in health policy programs implemented at school;
- provide students with access to clean water and allow them to drink water during class;
- encourage PA, including interclass activities, and active participation in physical education classes.

*Recommendations to regional authorities*

To be effective, prevention of obesity must be taken at various organizational levels. In addition to activities at the national level, all activities undertaken at the regional level are essential. The tasks of the regional authorities in the field of obesity prevention include:

- building public–private partnerships, engaging all entities to cooperate, in particular nongovernmental organizations promoting a healthy lifestyle, consumers, organizations, and private sector entities, including the food industry and media—joint activities to promote healthy behavior;
- use of mechanisms/instruments of impact available at a given level (including legal regulations), which may improve environmental conditions to those that are more conducive to pro-health behavior;
- implementation of health policy programs dedicated to the widest possible group of recipients; programs aimed, in particular, at building proper eating behavior and increasing PA; providing additional measures to children and adolescents from the group at higher risk of developing obesity and its complications;
- creating social campaigns aimed at improving diet and increasing PA;
- assistance in organizing psychological support;
- organization of events during which pro-health behaviors will be promoted (picnics, sports events involving whole families, educational workshops, culinary shows);
- investments in infrastructure supporting pro-health behavior (e.g., playgrounds with devices enabling the movement of the youngest playgrounds, catering studios in educational institutions, community centers, bicycle paths, walking paths, etc.), creating conditions for active recreation;
- cooperation with the scientific and medical community in the region for the best possible diagnosis of health needs and the provision of services corresponding to the diagnosed needs [217,218,219].

## Figures and Tables

**Table 1:** The most common gene mutations related to monogenic obesity and their characteristic features (besides of severe obesity of early origin).

Gene Name	Disorders Causing the Development of Obesity
Melanocortin 4 receptor	Somatomegaly, increased head circumference, hyperinsulinemia
Leptin	Hypogonadotropic hypogonadism, hypothyroidism, growth hormone deficiency, immune deficiency
Leptin receptor	Hypogonadotropic hypogonadism, hypothyroidism, growth hormone deficiency, immune deficiency
Single-minded homolog 1	Autism, behavioral problems
Kinase suppressor of Ras 2	Insulin resistance, low heart rate, reduced basal metabolic rate
Proopiomelanocortin	ACTH/adrenal insufficiency, abnormal pigmentation
Protein convertase subtilisin/kexin 1	Neonatal diarrhea, hypoglycemia, hypothyroidism, adrenal insufficiency, diabetes insipidus
Neurotrophic receptor tyrosine kinase 2	Developmental delay, hyperactivity
Melanocortin 2 receptor accessory protein 2	None
SH2B adapter protein 1	Severe insulin resistance, behavioral difficulties, and developmental delay
Brain-derived neurotrophic factor	Developmental delay, hyperactivity

**Table 2:** Clinical characteristics and results of blood tests in endocrine causes of obesity.

Endocrine Disorder	Other Clinical Characteristics	Lab Test Results
hypothyroidism	dry skin and hair, cold intolerance, face edema, bradycardia, delayed reflex timing, low blood pressure with small amplitude, delayed toothing, delayed puberty, goiter, constipation	↑ thyrotropin (TSH) ↓ free thyroxin (fT4) ↓ free triiodothyronine (fT3)
hypercortisolemia	moon face, central obesity, increased blood pressure, wide purple striae, muscular weakness, easy bruising, delayed puberty	↑ cortisol level in 1 mg dexamethasone test ↑ free cortisol in urine
growth hormone deficiency	visceral obesity, low muscle mass, younger than chronological age, appearance of the face, micropenis, delayed toothing	↓ growth hormone secretion in stimulation tests
pseudohypoparathyroidism	round face, short neck, shortening of the metacarpal and metatarsal bones (especially the 4th and 5th bones), features of hypocalcemia, subcutaneous ossification, intellectual impairment, delayed puberty, features of other hormonal disturbances (hypothyroidism, hypogonadism)	↓ calcium ↑ phosphorus ↑ parathyroid hormone (PTH)

**Table 3:** Dietary and other lifestyle modifications.

	Interventions
Interventions in eating behaviors	Eating five structured meals per day (three main and two complementary) without snacking/eating meals Every meal should contain protein, carbohydrates, and healthy fats Eating at regular times every 3–4 h Avoid skipping breakfast and meals at school. Eating fruits (2–3 portions a day), vegetables (at least 3 portions a day, green vegetables in plenty) Eating dairy products (not sweetened) minimum 2–3 portions a day Avoid consuming high-energy and low nutrient density foods (e.g., sweetened or energizing drinks, fast-food, high-energy snacks, e.g., chips, sticks, additives such as sauces, mayonnaise) Encouraging more water intake instead of sweetened beverages Encouraging the reading of food labels, especially regarding added sugars Encouraging the family to eat meals together as much as possible Limiting eating out, especially in fast-food restaurants Adjusting portion sizes appropriately for age Avoiding watching television, tablets, and smartphones while eating
Physical activity	Daily at least 60 min of moderate to vigorous aerobic PA vigorous intensity Limitation of screen time outside of school to 1–2 h daily Engaging in fun and age-specific exercise that is appropriate for the individual’s abilities
Behavioral interventions	Identifying disorders such as depression, eating disorders, body image problems, and anxiety

**Table 4:** Share of macronutrients in meal plan.

	Nutrient Intake	
	Obesity	Severe Obesity and/or Metabolic Complications
Carbohydrate *	45–65% kcal/day	45–50% kcal/day
Simple sugars *	<10% of total daily energy intake (unless the sugars are contained in fresh fruits and vegetables)	
Proteins	No less than 1 g/kg of actual body weight/day	
Fat *	20–35% of the diet (35–40% in toddlers)	20–30% of the diet (not less than 30% in toddlers)
Fibre	age (year) + 5–10 g/day	

* % of daily energy intake.

## Data Availability

Not applicable.
